# Phylogenetic studies of the genus *Cebus *(Cebidae-Primates) using chromosome painting and G-banding

**DOI:** 10.1186/1471-2148-8-169

**Published:** 2008-06-05

**Authors:** PJS Amaral, LFM Finotelo, EHC De Oliveira, A Pissinatti, CY Nagamachi, JC Pieczarka

**Affiliations:** 1 Universidade Federal do Pará. Laboratório de Citogenética, Belém, PA, Brazil; 2 CNPq Researcher, Brazil; 3 Centro de Primatologia do Rio de Janeiro – CPRJ/FEEMA, Brazil; 4 CAPES Masters Scholarship on Genetics and Molecular Biology, Brazil; 5 CNPq Masters Scholarship on Genetics and Molecular Biology, Brazil; 6 Instituto de Ciências Biológicas, Universidade Federal do Pará., Campus do Guamá, Av. Perimetral, sn. Guamá, Belém – Pará, Brazil

## Abstract

**Background:**

Chromosomal painting, using whole chromosome probes from humans and *Saguinus oedipus*, was used to establish karyotypic divergence among species of the genus *Cebus*, including *C. olivaceus*, *C. albifrons*, *C. apella robustus *and *C. apella paraguayanus*. Cytogenetic studies suggested that the species of this genus have conservative karyotypes, with diploid numbers ranging from 2n = 52 to 2n = 54.

**Results:**

Banding studies revealed morphological divergence among some chromosomes, owing to variations in the size of heterochromatic blocks. This analysis demonstrated that *Cebus *species have five conserved human associations (i.e., 5/7, 2/16, 10/16, 14/15, 8/18 and 3/21) when compared with the putative ancestral Platyrrhini karyotype.

**Conclusion:**

The autapomorphies 8/15/8 in C. albifrons and 12/15 in *C. olivaceus *explain the changes in chromosome number from 54 to 52. The association 5/16/7, which has not previously been reported in Platyrrhini, was also found in *C. olivaceus*. These data corroborate previous FISH results, suggesting that the genus *Cebus *has a very similar karyotype to the putative ancestral Platyrrhini.

## Background

Taxonomy of the genus *Cebus *is a controversial subject. Members of this genus display intense variations in fur color and pattern depending on age, gender and geographical location [1]. Despite these variations, most authors agree that *Cebus *comprises five species: *C. apella*, *C. albifrons*, *C. capucinus, C. olivaceus *and *C. kaapori *[2-5]. Groves (2001) [6] published a new taxonomy for the genus, where he recognizes four species with subspecies: *Cebus apella *(*C. a. apella*, *C. a. fatuellus*, *C. a. macrocephalus*, *C. a. peruanus*, *C. a. tocantinus *and *C. a. margaritae*), *C. libidinosus *(*C. l. libidinosus*, *C. l. pallidus*, *C. l. paraguayanus *and *C. l. juruanus*), *C. nigritus *(*C. n*. *nigritus*, *C. n. robustus *and *C. n. cucullatus*) and *C. xanthosternos*. Silva Júnior (2002) [7] classification has some differences. For instance, Groves (2001) [6] recognizes *robustus *as a subspecies of *C. nigritus *while Silva Junior (2002) [7] recognizes it as a full species.

Cytogenetic studies on *Cebus *have shown that the diploid number ranges from 52 to 54 chromosomes. The species of this genus have large blocks of constitutive heterochromatin, mainly found in interstitial and distal regions, which displays intraspecific variation, few biarmed chromosomes and secondary constrictions in two acrocentric pairs [8-12]. To date, human chromosomal painting has been used to analyze three species of *Cebus*: *C. capucinus *[13]*C. apella *[14] and *C. nigrivittatus *[15].

Interspecies chromosomal comparisons of *Cebus *have been performed using G- and Q-banding patterns. These comparisons [16] suggest that *C. capucinus*, *C. albifrons *and *C. apella *share 19 chromosome pairs, *C. capucinus *and *C. albifrons *share 25 pairs and *C. capucinus *and *C. apella *share 20 pairs. Among these three species, the karyotype of *C. capucinus *most resembles the putative ancestor, as all chromosomes found in *C. capucinus *are observed in *C. albifrons *and *C. apella*. Furthermore, *C. albifrons *and *C. apella *seem to have been independently derived from an ancestor with a karyotype similar to *C. capucinus*. The *C. capucinus *karyotype is closer to *C. albifrons *than to *C. apella*.

Zoo-FISH comparative chromosome painting is a powerful method for detecting chromosome homologies between species and for resolving phylogenetic controversies. This study compared the chromosome homologies present among *Cebus apella paraguayanus *(2n = 54), *Cebus apella robustus *(2n = 54), *Cebus albifrons *(2n = 52) and *Cebus olivaceus *(2n = 52) using G-banding and chromosome painting with whole chromosome probes derived from humans and *Saguinus oedipus*. Our results were compared with previous reports to propose a phylogeny for these species, using chromosomal characters in a parsimony analysis.

## Methods

Metaphasic chromosomes from four *Cebus *taxa (Table 1) were obtained by lymphocyte [17] and fibroblast culture. Karyotypes were organized following the protocol of Matayoshi et al. (1986) [18].

**Table 1:** *Cebus *samples used in this research.

Taxon	2n	Number of animals and gender	Cell culture	Institution
*C. a. paraguayanus*	54	1 male and 1 female	Lymphocytes	Passeio Público (Curitiba-PR)
*C. a. robustus *(Kuhl, 1820)	54	3 males	Lymphocytes and fibroblasts	Centro de Primatologia do Rio de Janeiro (Guarapimirim-RJ)
*C. albifrons *(Humboldt, 1812)	52	6 males	Lymphocytes and fibroblasts	Rio Branco-AC e Centro Nacional de Primatas (Ananindeua-PA)
*C. olivaceus *(Cuvier, 1819)	52	6 males	Lymphocytes and fibroblasts	Centro Nacional de Primatas (Ananindeua-PA) e Parque Zoobotânico Gavião Real (Capitão-Poço-PA)

G-banding was performed using the methods of Seabright (1971) [19]. FISH experiments were performed in all species using *S. oedipus *whole chromosome probes [20] and 24 different whole chromosome probes taken from humans (1–22 autosomes, X and Y). Probes were organized into four pools (H1-H4 and S1-S4, for human and *S. oedipus *chromosome paints, respectively) as previously described [21,20]. The probes were then labeled by DOP-PCR [22] using biotin-dUTP, digoxigenin-dUTP (Roche) and TAMRA-dUTP (Applied Biosystems/PE). *In situ *hybridization and detection were performed using the protocols of Neusser et al. (2001) [21] and De Oliveira et al. (2005) [23]. Nomenclatures of chromosomes and chromosome segments were consistent with Neusser et al. (2001) [21] and De Oliveira et al. (2005) [23]. Human and *S. oedipus *probes were applied to all of the taxa except *C. olivaceus*, which was analyzed solely using human probes.

G-banded metaphases were captured using a Zeiss III photomicroscope with Imagelink HQ film manufactured by Kodak. FISH/DAPI metaphases were captured with a CCD camera under a Zeiss Axiophot microscope. Images were analyzed using Adobe Photoshop 7.01.

Phylogenetic analysis was performed by applying a cladistic method with parsimony criteria. A basic data matrix was built by comparing chromosomal differences among species, as determined by FISH or G-banding. Previously reported chromosome painting data was used for *C. apella sp*. [14] and *C. capucinus *[13]. *Saimiri sciureus *and *Callithrix jacchus *were used as outgroups. PAUP (Phylogenetic Analysis Using Parsimony, 4.0b1 for Microsoft Windows) software was used to build the cladogram, which was then tested using the bootstrap method [24].

## Results

### Cytogenetic analysis

Diploid number analysis confirmed a range of 52 to 54 chromosomes in the genus *Cebus*. *Cebus apella paraguayanus *and *C. a. robustus *had 54 chromosomes (10 pairs were biarmed and 16 pairs were one-armed). *Cebus albifrons *had 52 chromosomes (9 pairs were biarmed and 16 pairs were one-armed), as did *C. olivaceus *(10 pairs were biarmed and 15 pairs were one-armed). Sex chromosomes were similar in all the species, with submetacentric X chromosome and a small acrocentric chromosomes Y chromosome.

**Figure 1:**
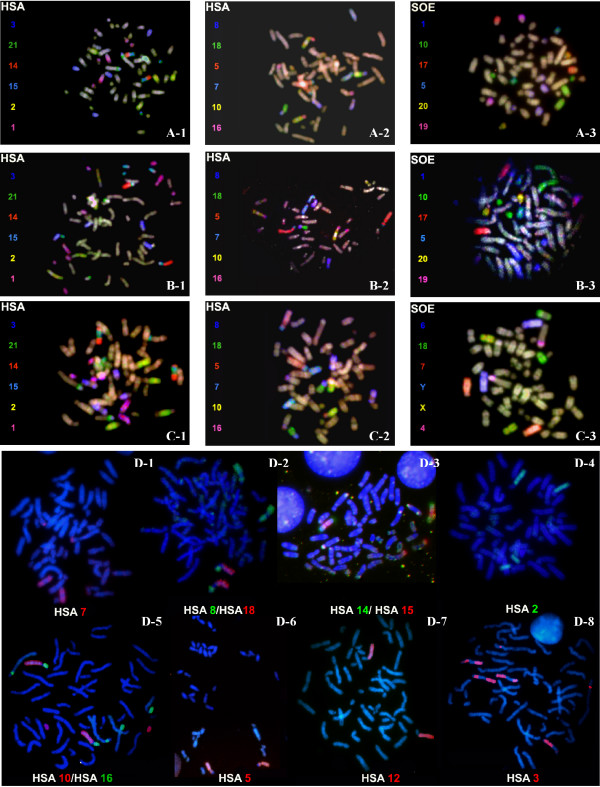
**Representative multi-color FISH experiments using human (HSA), *S. oedipus *(SOE) and painting probe sets to (A-1; A-2; A-3) *C.a. paraguayanus*, (B-1; B-2; B-3) *C.a. robustus*, (C-1; C-2; C-3) *C. albifrons*, and (D-1 to D-8) *C. olivaceus *metaphases.** Respective probe compositions and false color assignments are given beside each metaphase.

With the exception of the human Y chromosome paint, all human and *S. oedipus *probes painted chromosomes of *C. a. paraguayanus *(CAP), *C. a. robustus *(CAR), *C. albifrons *(CAL). Constitutive heterochromatin regions did not show any signal of hybridization on any of the species. Representative results from FISH experiments with human probes are shown in Figures 1A1;A2;B1;2;C1;C2 and D(1 to 8), while experiments using *S. oedipus *probes are shown in Figures 1A3;B3 and 1C3.

#### Cebus apella paraguayanus and Cebus apella robustus

*Cebus apella paraguayanus *and *C. a. robustus *displayed similar karyotypes. Human probes revealed 34 homologous segments. Synteny was conserved in 12 human chromosomes: HSA 4, HSA 5, HSA 6, HSA 9, HSA 11, HSA 12, HSA 13, HSA 17, HSA 19, HSA 20, HSA 22 and HSA X, which corresponded to CAP 2, CAP 1, CAP 3, CAP 18, CAP16, CAP 12, CAP 17, CAP 21, CAP 9, CAP 10, CAP 24 and CAP X in *Cebus*, respectively. Human associations commonly found in other species of Platyrrhini were found in these taxa, including 2b/16b, 3a/21, 5/7a, 8a/18, 10a/16a and 14/15a. SOE painting probes revealed 27 homologous segments. Eighteen conserved segments were identified between these *C. apella *subspecies and *S. oedipus*.

**Figure 2:**
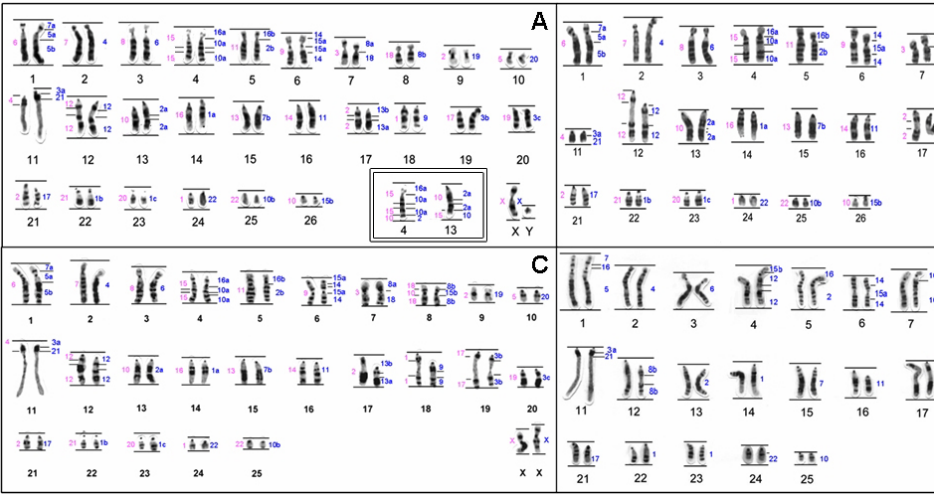
**Comparison of G-banding patterns observed between taxa of the genus *Cebus*, showing homologies found in CAPp = *C. a. paraguayanus*; CAPr = *C. a. robustus*; CAL = *C. albifrons *e CGR = *C. olivaceus*.** A, pericentric inversion; B, paracentric inversion; C, centric fusion/fission; D, amplification or deletion of the heterochromatic block.

**Figure 3:**
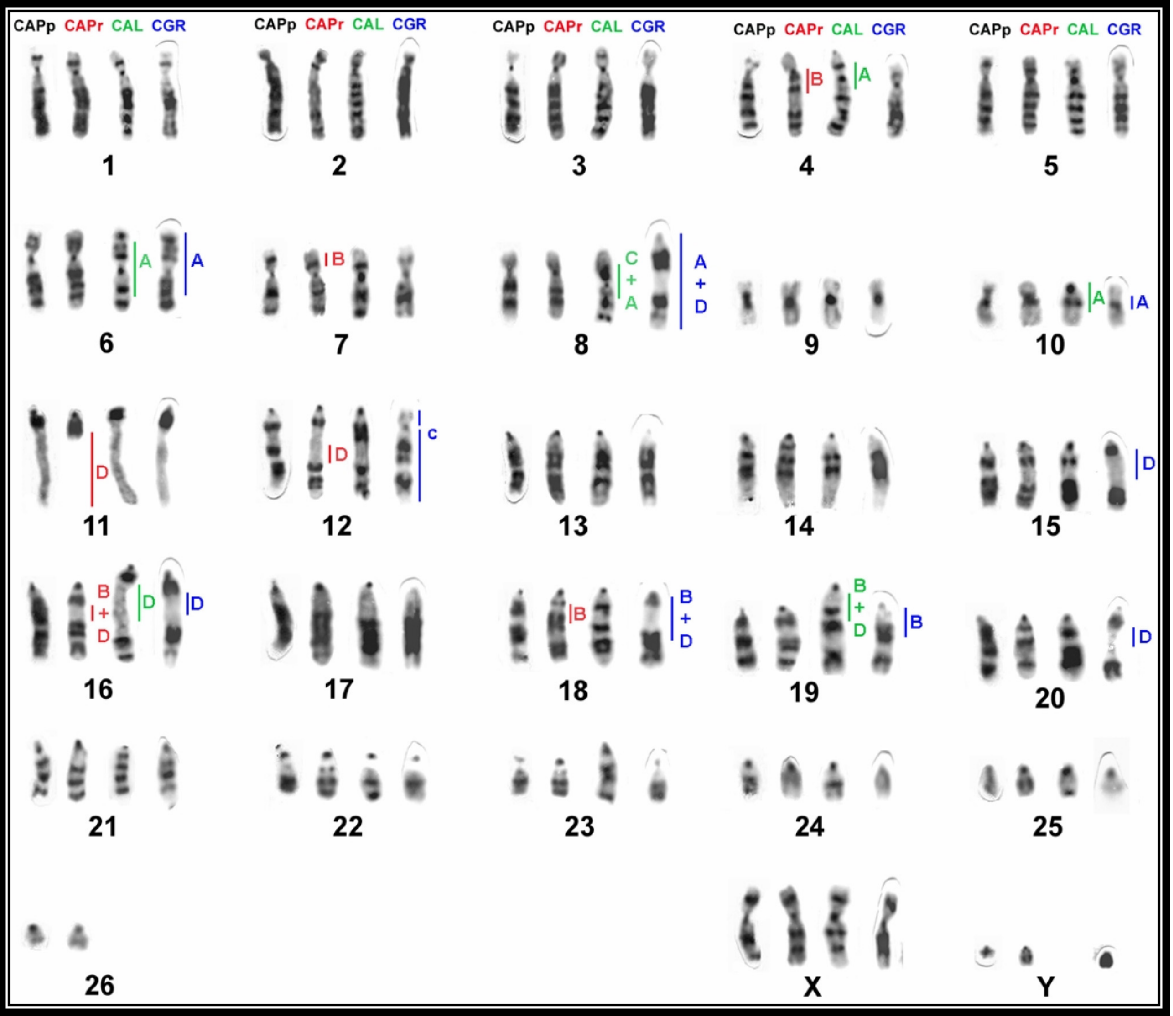
**Map of human chromosomes (blue, right side) and *S. oedipus *(red, left side) in the G-banded karyotypes of *C.a. paraguayanus *(A), *C.a. robustus *(B), *C. albifrons *(C) and the human probes in *C. olivaceus *(D).** Some syntenic groups have a small letter (e.g., a, b or c), according to the pattern established by Neusser *et al*. (2001). Groups without this letter correspond to whole chromosomes or chromosome segments that differed from those described by Neusser *et al*.

The SOE X and Y probes hybridized to the respective sex chromosomes of both *Cebus *species. The G-banded karyotype of *C. a. paraguayanus*, together with a summary of the chromosome painting results, is shown in Figure 2A. Figure 2B shows the karyotype of *C. a. robustus*. The *C. a. paraguayanus *male displayed a reciprocal translocation of segments homologous to HSA 2a e 10a, which was confirmed using the SOE 10 and SOE 15 probes (Figure 2A, inset). The two species differed in the distribution of constitutive heterochromatin, where the most obvious difference was the absence of the distal heterochromatic block in chromosome 11 of *C. a. robustus *(Figure 3).

#### Cebus albifrons

Results of this study revealed the conservation of 11 human chromosomes (i.e., HSA 4, HSA 6, HSA 9, HSA 11, HSA 12, HSA 13, HSA 17, HSA 19, HSA 20, HSA 22 and X), corresponding to *C. albifrons *chromosomes CAL 2, CAL 3, CAL 18, CAL 16, CAL 12, CAL 17, CAL 21, CAL 9, CAL 10, CAL 24 and CAL X. Four human chromosomes (i.e., HSA 5, HSA 14, HSA 18 and HSA 21) hybridized to a single chromosome of *C. albifrons*, but associated with other segments. We found seven human associations that were previously identified in Platyrrhini (i.e., 2b/16b, 3a/21, 5/7a, 8a/18, 8b/15b, 10a/16a and 15/14a/15a/14a). *S. oedipus *probes revealed 26 homologous segments (Figures 1C.1-C.3).

#### Cebus olivaceus

Results of this study revealed the conservation of nine human chromosomes (i.e., HSA 4, HSA 6, HSA 9, HSA 11, HSA 13, HSA 17, HSA 19, HSA 20 and HSA 22), corresponding to *C. olivaceus *chromosomes CGR 2, CGR 3, CGR 18, CGR 16, CGR 17, CGR 21, CGR 9, CGR 10 and CGR 24. We did not use human sex chromosome probes on this species. Four human probes (i.e., HSA 5, HSA 12, HSA 14 and HSA 18) hybridized to a single chromosome of *C. olivaceus*, but associated with other segments. Seven associations were found: 2/16, 3/21, 5/16/7, 8/18, 10a/16, 14/15/14 and 15/12 (Figures D-1 to D-8). Of these, 5/16/7 and 15/12 corresponded to autapomorphies.

#### Comparative analysis and phylogeny

**Figure 4:**
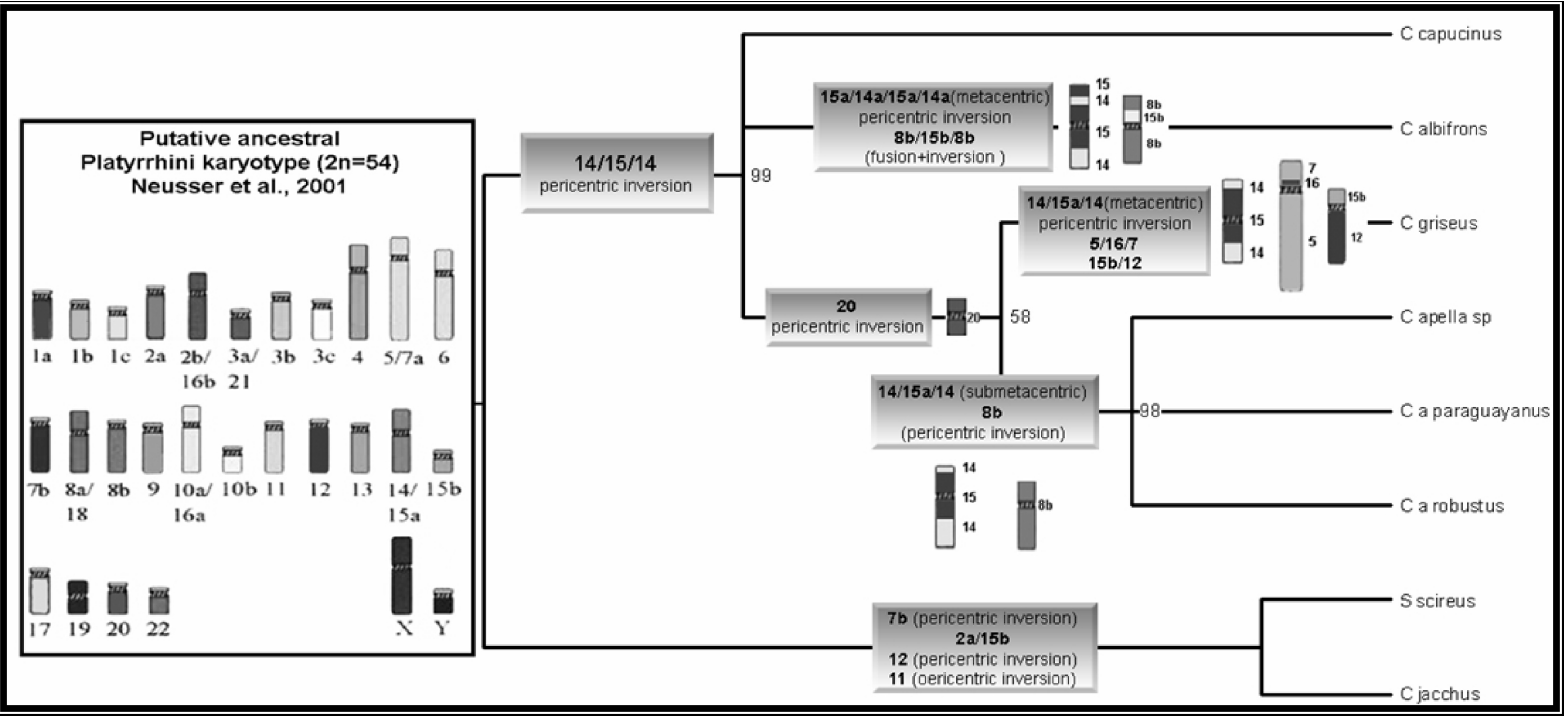
**Cladogram obtained from an analysis of chromosome painting and G-banding data in *Cebus *taxa, with 62 steps consistency index (CI) = 0.968, retention index (RI) = 0.926 and homoplasy index (HI) = 0.032.***S. sciureus *and *C. jacchus *were outgroups.

Human and *Saguinus oedipus *painting results, in combination with the G-banding results, allowed for a detailed comparison of chromosome homologies among members of the genus *Cebus*. A basic data matrix (BDM) was built using alternative forms of the chromosomes as characters (Table 2). These characters were then used in a binary matrix (Table 3). In this matrix the data of *Cebus apella sp *are from Garcia et al. (2000) [14] and of *C. capucinus *are from Richard et al. (1996) [13]. PAUP analysis resulted in a single cladogram (Figure 4) with 62 steps, a consistency index of 0.968, a retention index of 0.926 and a homoplasy index of 0.032.

**Table 2:** Chromosomal rearrangements related to each character found in the binary matrix.

1	5/7a	11	2b/16b (pi1)	21	2a (pi)	31	15b (free, A)	41	8b (pi)	51	9 (A)
2	5/16/7a	12	2b/16b (dis 2b1 and 2b2)	22	15a1/14	32	22 (pi)	42	8b/15b/8b	52	9 (pi)
3	5b/5a/16	13	10b (free, A)	23	9/14/15/14/15/14/15	33	22 (A)	43	12 (A)	53	1a (dis1a1 and 1a2)
4	7b (A)	14	2a/10b	24	20 (A)	34	3a/21 (A)	44	12 (pi)	54	1a (pi)
5	7b (pi)	15	10a/1a	25	20 (pi)	35	3a/21 (M pi)	45	12/15b	55	1a (A)
6	4 (pi)	16	2a (A)	26	20/17/13b	36	3b (A)	46	19 (A)	56	1b (pi)
7	10a/16a	17	2a/15b	27	20/17	37	3b (pi)	47	19 (pi)	57	1b (A)
8	10a/16a (pi2)	18	14/15a/14 (SM)	28	13a/9/22	38	3b/10b	48	19/1b	58	1b/19
9	10a/16a/2	19	14/15a/14 (pi)	29	13b/17	39	3c/20	49	11 (A)	59	13 (dis13a and 13b)
10	2b/16b	20	14/15a/14 (pa)	30	13b/17 (pa)	40	8b (A)	50	11 (pi)	60	7b (A)
										61	7b (pi)

pi = pericentric inversion; pa = paracentric inversion; dis = dissociation; M = metacentric chromosome; SM = submetacentric chromosome; A = acrocentric chromosome.

**Table 3:** Binary character matrix used for the cladistic analysis (0 = absence of the character, 1 = presence of the character).

**Char**.	CAPsp	CAPp	CAPr	CCA	CAL	CGR	SSC	CJA
**1**	1	1	1	1	1	0	1	1
**2**	0	0	0	0	0	1	0	0
**3**	0	0	0	0	0	0	1	0
**4**	1	1	1	1	1	1	0	0
**5**	0	0	0	0	0	0	1	1
**6**	0	0	0	0	0	0	1	0
**7**	1	1	1	1	1	1	0	1
**8**	0	0	0	0	0	0	1	0
**9**	0	1	0	0	0	0	0	0
**10**	1	1	1	1	1	1	1	0
**11**	0	0	0	0	0	0	1	0
**12**	0	0	0	0	0	0	0	1
**13**	1	1	1	1	1	1	0	0
**14**	0	1	0	0	0	0	0	0
**15**	0	0	0	0	0	0	0	1
**16**	1	1	1	1	1	1	0	1
**17**	0	0	0	0	0	0	1	1
**18**	1	1	1	0	0	0	0	0
**19**	0	0	0	1	1	1	0	0
**20**	0	0	0	0	1	0	0	0
**21**	0	0	0	0	0	0	1	0
**22**	0	0	0	0	0	0	0	1
**23**	0	0	0	0	0	0	1	0
**24**	0	0	0	1	1	0	1	1
**25**	1	1	1	0	0	1	0	0
**26**	0	0	0	0	0	0	0	1
**27**	0	0	0	0	0	0	0	1
**28**	0	0	0	0	0	0	0	1
**29**	0	0	0	0	0	0	0	1
**30**	0	0	0	0	0	0	0	1
**31**	1	1	1	1	0	0	0	0
**32**	0	0	0	0	0	0	1	0
**33**	1	1	1	1	1	1	0	1
**34**	1	1	1	1	1	1	1	0
**35**	0	0	0	0	0	0	0	1
**36**	1	1	1	1	1	1	0	1
**37**	0	0	0	0	0	0	1	0
**38**	0	0	0	0	0	0	1	0
**39**	0	0	0	0	0	0	1	0
**40**	0	0	0	1	1	1	1	1
**41**	1	1	1	0	0	0	0	0
**42**	0	0	0	0	1	0	0	0
**43**	1	1	1	1	1	1	0	0
**44**	0	0	0	0	0	0	1	1
**45**	0	0	0	0	0	1	0	0
**46**	0	0	0	0	0	0	1	0
**47**	1	1	1	1	1	1	0	1
**48**	0	0	0	0	0	0	1	0
**49**	1	1	1	1	1	1	0	0
**50**	0	0	0	0	0	0	1	1
**51**	1	1	1	1	1	1	1	0
**52**	0	0	0	0	0	0	0	1
**53**	0	0	0	0	0	0	0	0
**54**	0	0	0	0	0	0	1	0
**55**	1	1	1	1	1	1	0	1
**56**	0	0	0	0	0	0	1	0
**57**	1	1	1	1	1	1	0	1
**58**	0	0	0	0	0	0	1	0
**59**	0	0	0	0	0	0	0	1
**60**	1	1	1	1	1	1	0	0
**61**	0	0	0	0	0	0	1	1

*Cebus apella sp*., CAPsp *C. a. paraguayanus*, CAPp;*C. a. robustus*, CAPr; *C. capucinus*, CCA;*C. albifrons*, CAL; *C. olivaceus*, CGR; *Saimiri sciureus*, SSC; *Callithrix jacchus*, CJA.

## Discussion

Many reports use morphological [25-28], molecular [29-33] or chromosomal [34,35,21] data to dissect the phylogenetic relationships among New World monkeys at the family level. However, ordering the species below the genus level is a fundamental step toward reorganizing the phylogenetic relationships among these taxa. This realization prompted us to analyze taxa from the genus *Cebus *to better understand their chromosomal divergences and to clarify their phylogenetic positions.

All of the syntenies in the putative ancestral Platyrrhini karyotype (i.e., 3a/21, 5/7a, 2b/16b, 8a/18, 14/15a e 10a/16a) were conserved in the *Cebus *species, consistent with previous reports on *C. apella sp*. [14] and *C. capucinus *[13]. Our data in *C. a. paraguayanus *are quite similar with the one described by Garcia et al. (2000) [14] on *C. apella sp*. Interestingly, the 5/7a association was found in all but one member of the genus *Cebus*. In *C. olivaceus*, this association possessed an additional segment homologous to HSA16. This segment probably fused in tandem with chromosome HSA7, followed by a paracentric inversion that resulted in the association CGR 7/16/5, which has not been reported before in New World primates. The association 5/16 is found in *Saimiri *but not in the other members of the genus *Cebus*. However, the segment homologous to HSA16 has different sizes in these species, which strongly supports the occurrence of a homoplasy. Garcia et al. (2002) [15] described human chromosomal painting in *C. nigrivittatus*, a synonymy for *C. olivaceus*. They did not find the HSA16 fragment in pair 1 and also the morphology of pair 10 is slightly different, with a short arm in our sample. This can be a consequence of a pericentric inversion or a heterochromatin heteromorphism.

Associations 2b/16b and 8a/18 were present without any alterations in members of the genus *Cebus*. The association 14/15a is inverted in all *Cebus *species, revealing a synapomorphy. In *C. a. paraguayanus *and *C. a. robustus*, this association exists in a submetacentric pair. In *C. albifrons*, this association exists in a metacentric chromosome due to a second inversion that gave rise to the association 15a/14/15a/14. A different inversion of HSA15a was found in *C. olivaceus*, changing the morphology of HSA15a from acrocentric to metacentric.

Associations 12/15 in *C. olivaceus *and 8/15/8 in *C. albifrons*, which was confirmed by the *S. oedipus *association 18/10/18, explain the reduction in diploid number from 54 to 52. An in tandem fusion, followed by a pericentric inversion, occurred in *C. albifrons*. Conversely, a Robertsonian rearrangement occurred in *C. olivaceus*.

Chromosomal data were used to obtain a cladogram that reconstructed a possible sequence of chromosome rearrangements leading to karyotypical differentiation into the *Cebus *genus (Figure 4). The cladogram supports the notion that the monophyly of *Cebus. C. apella sp*., *C. a. paraguayanus *and *C. a. robustus *are closely related, sharing two synapomorphic traits (i.e., the association 14/15/14 that resulted in a submetacentric chromosome and the pericentric inversion that corresponded to the HSA8b probe). *C. capucinus *occupies a more basal position, with a chromosomal composition very similar to the putative ancestral Platyrrhini karyotype, consistent with previous reports by Richard et al. (1996) [13]. The phylogenetic relationships of *C. capucinus *and *C. albifrons*, in relation to the ancestral karyotype, are not clearly defined. However, it is clear that the karyotype of *C. albifrons *differs from that of *C. capucinus *by a pericentric inversion in the 14/15 association, which results in a metacentric association 15/14/15/14. We also identified an in tandem fusion, followed by a pericentric inversion involving the homologous human chromosomes HSA15b and HSA8b, in *C. albifrons*. As *C. olivaceus *is closely related to *C. apella*, these species share the chromosomal inversion homologous to HSA20. Differentiation between *C. olivaceus *and *C. apella *is possible via a pericentric inversion in the association 14/15/14 and a Robertsonian rearrangement in the chromosomes homologous to HSA12 and HSA15b on their Figure 2c. Garcia et al. (2002) [15] left open two possibilities for chromosome 6 in the *Cebus *ancestral karyotype: the ancestral form could be a metacentric like in CAL, CCA, and CNI karyotypes or a submetacentric like in CAP. Our cladistic analysis has shown that the ancestral form is the metacentric.

This study used chromosome painting in conjunction with G-banding to confirm the ability of these techniques to generate consistent and reliable data. These data were interpreted using a cladistic analysis capable of generating a cladogram with a high degree of consistency. Future studies should use molecular markers to further explore the phylogeny described here.

## Conclusion

Chromosome painting in several species of *Cebus *allowed us to define all the rearrangements that ocurred during its speciation. It was also possible to use FISH and G-banding data, both from our results and from literature, to build a cladogram that reconstructed a possible sequence of chromosome rearrangements leading to karyotypical differentiation into the *Cebus *genus.

## Authors' contributions

PJSA carried out chromosome painting in *Cebus olivaceus*, organized the data and wrote most of the paper. LFMF carried out chromosome painting in *Cebus albifrons *and contributed to the discussion of data. EHCO carried out chromosome painting in *C. a. robustus *and contributed to the discussion of data. AP collected the samples, classified the species and discussed the phylogenetic implications of the data. CYN carried out chromosome painting in *Cebus apella paraguayanus *and performed the cladistic analysis. JCP conceived of the study, participated of the techniques development and coordinated the study. All authors read and approved the final manuscript.
